# Insights into the Properties and Potential Applications of Renewable Carbohydrate-Based Ionic Liquids: A Review

**DOI:** 10.3390/molecules25143285

**Published:** 2020-07-20

**Authors:** Bartłomiej Gaida, Alina Brzęczek-Szafran

**Affiliations:** Department of Chemical Organic Technology and Petrochemistry, Silesian University of Technology, 44100 Gliwice, Poland; bartlomiej.gaida@polsl.pl

**Keywords:** carbohydrate, sugar, ionic liquid, biomass, bio-ILs, biodegradable, low toxicity

## Abstract

Carbohydrate-derived ionic liquids have been explored as bio-alternatives to conventional ionic liquids for over a decade. Since their discovery, significant progress has been made regarding synthetic methods, understanding their environmental effect, and developing perspectives on their potential applications. This review discusses the relationships between the structural properties of carbohydrate ionic liquids and their thermal, toxicological, and biodegradability characteristics in terms of guiding future designs of sugar-rich systems for targeted applications. The synthetic strategies related to carbohydrate-based ionic liquids, the most recent relevant advances, and several perspectives for possible applications spanning catalysis, biomedicine, ecology, biomass, and energy conversion are presented herein.

## 1. Introduction

Dynamic economic growth and an expanding population have contributed to increased consumption of fossil fuels and derived chemicals, thereby also highlighting the environmental effects of their manufacture, exploitation, and utilization [1]. Increasing global environmental consciousness has inspired the development of sustainable and eco-friendly processes, particularly in a chemical industry with a notable interest in molten salts. Ionic liquids (ILs), which are composed of organic cations and organic or inorganic anions, show negligible vapor pressure, high thermal stability, and very low flammability. These properties initially classified them as “green” solvents, representing them a promising alternative to VOCs [2,3]. However, the enthusiasm towards ILs that was expressed at the beginning of the 21st century began to decrease when people started considering the economic and environmental issues related to their production and use. Ionic liquids exhibit high chemical stability and often high solubility in water, so issues linked with their release into the environment, where they could accumulate and persist for a long time, have warranted investigations [4]. Nevertheless, significant progress in the field of IL research in recent years, and the continuing efforts to push the boundaries, have shown that ILs have clear industrial potential. In fact, an increasing number of processes are operating successfully thanks to IL technology (e.g., BASIL™, HycaPure Hg, and Ioncell). Prices for some ILs decline with greater economies of scale, and their ecotoxicology and biodegradability are significantly improved [5]. The latter aspect has been highly influenced by the development of biomass-derived ILs that originate from compounds commonly existing in nature such as amino acids, carbohydrates, carboxylic acids, or choline. Biomass-derived ILs (bio-ILs) demonstrate lower toxicity and higher biocompatibility than their fossil fuel-derived analogues and have recently been reviewed in detail by Gomes et al. [6] and Hulsbosch et al. [7]. 

Among bio-ILs, considerable progress has recently been made regarding the use of sugars or carbohydrates, which are advantageous because they are abundant, inexpensive, renewable, and environmentally friendly. Ionic liquids and salts produced using carbohydrates have been investigated for over a decade, with initial efforts focused on their functionalization and how the introduced functionalities relate to their physicochemical characteristics [8]. Notably, such systems have been recognized for their applicability as organocatalysts and solvents in ways that take advantage of their chirality [9]. Currently, broadened perspectives regarding potential IL applications span electrochemistry [10], energy storage [11,12], anti-bacterial systems [13,14], herbicides [15], and biomass conversion [16]. Carbohydrates have also recently been shown to reduce toxicity when incorporated into common imidazolium IL structures, thereby further enhancing their biocompatible character [17].

Although sugars are an economically favorable starting material, the process of manufacturing their ionic derivatives was originally complicated because of the required multiple-step synthetic method. Therefore, recent research efforts aimed at improving and simplifying the synthesis of carbohydrate-derived ILs have focused on implementing protocols to reduce the number of synthetic steps and/or integrate green chemistry approaches [11,17,18]. Such improvements have highlighted a more economically promising perspective regarding carbohydrate-based ILs and their applications.

Herein, we discuss the influence of the structure of carbohydrate ILs and salts on their environmental impact and physiochemical properties. The most common synthetic protocols are presented, along with comprehensive perspectives on the possible applications of these ionic liquids and salts.

## 2. Synthesis of Carbohydrate-Derived ILs and Salts

The carbohydrate-derived IL family has grown considerably since 2003 when Handy et al. [19] presented the synthesis of an imidazolium IL developed from fructose. Although the final IL did not contain the carbohydrate moiety in its structure, it was the first time that sugars were used as a renewable starting material for the preparation of ILs. About one year later, the first carbohydrate-derived IL was obtained, where d-glucopyranoside was transformed into the corresponding cation [20]. Since these reports, a wide range of ILs have been developed based on carbohydrates and their derivatives including glucose [8,21,22,23,24,25], galactose [26,27], fructose [19], ribose [26], xylose [27,28], arabinose [29], isomannide [30,31,32], isosorbide [33,34], ribitol [35], and mannitol [25]. The syntheses of these ILs have already been reviewed by Chiappe et al. [36], Kaur et al. [9], and Hulsbosch et al. [7]. Therefore, this review briefly presents the most common synthetic pathways for obtaining sugar-based ILs to provide a relevant context for various recently reported examples of ILs and primarily discusses new advances and promising strategies.

**Figure 1:**
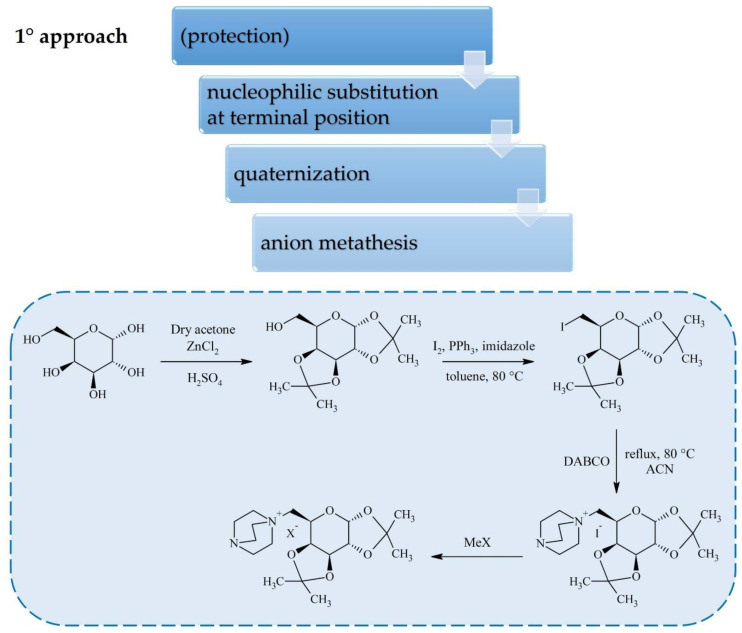
Synthesis of carbohydrate-based ionic liquids (ILs) via modification of d-galactose at terminal position.

**Figure 2:**
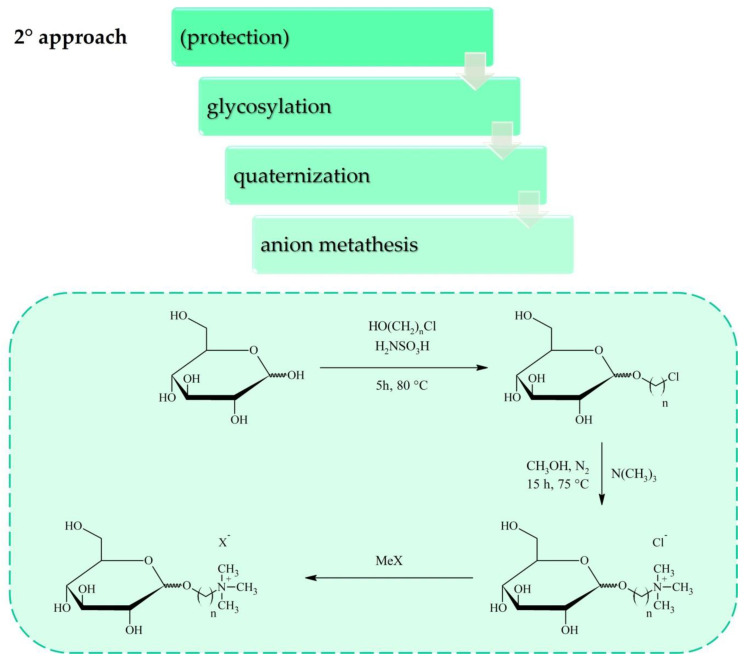
Synthesis of ILs with carbohydrate-based cation via modification of d-glucose at anomeric position.

**Figure 3:**
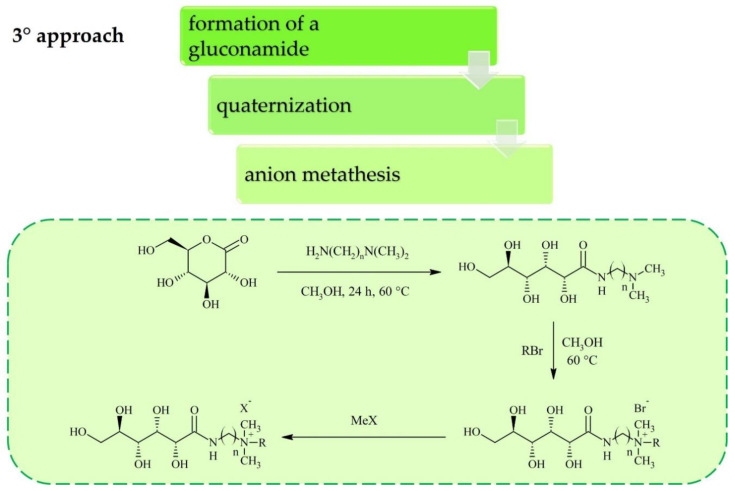
Synthesis of ILs with carbohydrate-based cation via modification of glucono-δ-lactone.

**Figure 4:**
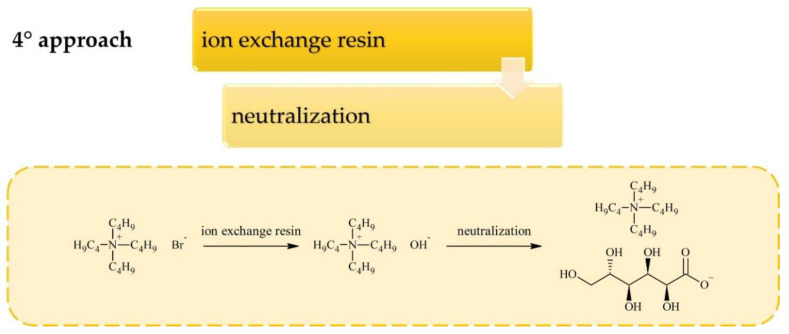
Synthesis of ILs with carbohydrate-based anion.

**Figure 5:**
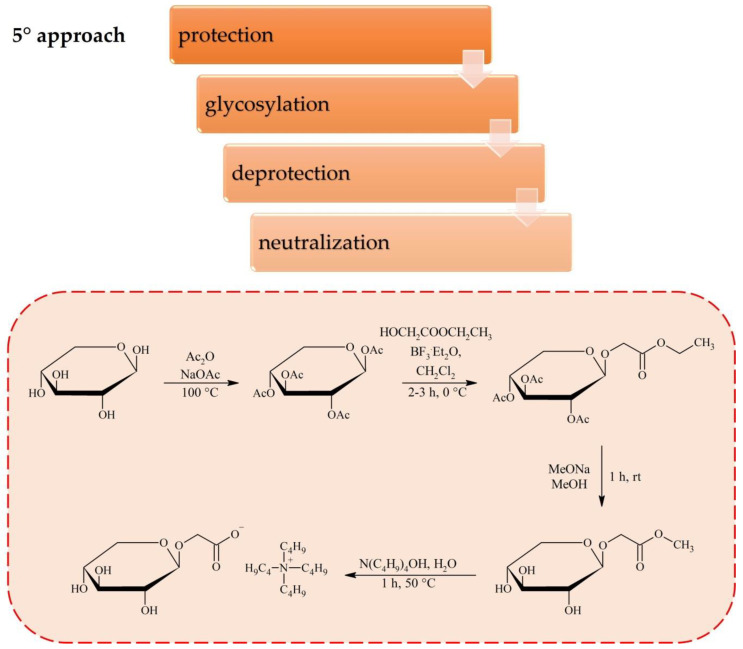
Synthesis of ILs with carbohydrate-based anion via modification of d-xylose at the anomeric position.

Carbohydrates can be converted into cations or anions for ILs through standard reactions that are already applied widely in carbohydrate chemistry. Most of the research on sugar-based ILs has focused on cation synthesis which can be achieved according to one of the general procedures depicted in Figure 1, Figure 2 and Figure 3. Although the conversion of sugar moieties into cations has been widely explored, the most promising synthetic route towards ILs (in terms of the number of required synthetic steps) involves transforming carbohydrates into anions (Figure 4 and Figure 5) such as gluconate, glucuronate, or galacturonate [12,22,37,38]. Moreover, functionalization of poly(ionic liquid)s (PILs) with sugar moieties has also recently been investigated [13,14]. 

In the first approach, carbohydrates are used as starting materials in unprotected cyclic forms or as glucosides and some synthetic strategies require protection of the hydroxyl groups by etherification or acetylation (mostly to facilitate a later purification step). Such intermediates are prone to nucleophilic substitution at the terminal position, followed by quaternization with aliphatic or aromatic amines, like pyridine or 1,4-diazobicyclo[2.2.2]octane (DABCO), to yield halide salts. The physiochemical properties of as-prepared ILs and salts can be further tuned via anion exchange.

Reiβ et al. [39] recently explored various functionalization possibilities in ILs based on pentoses (d-ribose, d-lyxose, d-xylose, d-arabinose) and hexoses (d-glucopyranose). Generally, in these reactions, the pentoses are peracetylated, with subsequent substitution of a thiophenyl group at the anomeric center. This site is then further reduced using tributyltin hydride to yield the corresponding 1-deoxypentose. In the next step, the acetyl groups are deprotected, and the primary OH group is tritylated in order to transform the remaining secondary hydroxyl groups into methyl, ethyl, propyl, or allyl ethers. Finally, the unprotected OH group at C5 is converted into a triflate moiety and simultaneously quaternized with pyridine. Similarly, methyl, allyl, and phenyl β-d-gdlucosides were employed in an analogous synthetic procedure; however, in these cases, acetylation and reduction at the anomeric positions were not performed. The overall yields of pentose- and hexose-based ILs were 25–32% (8 steps) and 31–56% (5 steps), respectively. 

Kaur et al. [40] used d-galactose as a starting material to obtain a series of carbohydrate ILs with various anions (Figure 1). In this method, the secondary hydroxyl groups of d-galactose are protected with acetone in the presence of ZnCl_2_ and H_2_SO_4_, and the remaining primary OH group is substituted with iodide. After purification by column chromatography, the isolated derivative is quaternized using DABCO. Different sodium and potassium salts have been used for anion metathesis, eventually producing ILs with [I]^−^, [BF_4_]^−^, [PF_6_]^−^, [BrCH_2_CH_2_SO_3_]^−^, [CF_3_SO_3_]^−^, or [SbF_6_]^−^ anions. The overall yield for this 4 step synthesis is 32–35%, while yields of individual steps were between 65% and 82%. Synthesis of ILs according to this procedure has also been carried out in our group using methyl-α-d-glucopyranoside as a starting material and without the protection step what increased overall yields to 44–65% for the resulting hydrogen-bond-rich ILs [12]. 

A second synthetic procedure is based on modification of the carbohydrate unit at the anomeric carbon (Figure 2). Chrobok’s group has applied this method to study the glycosylation of d-glucopyranose with halogenoalcohols (e.g., bromoethanol, chloroethanol, 3-chloropropanol, or 3-chloro-1,2-propanediol), generating glycosides with yields of 50% to 78% after purification by column chromatography [12,15,41,42]. Glycosides can be further quaternized with amines to yield halide salts, which can be applied as task-specific ILs after metathesis with specific anions, such as [NTf_2_]^−^ [12,41], [MCPA]^−^, [2,4-D]^−^ [15], [N(CN)_2_]^−^ [12], or amino acid anions [42]. It has also been reported that triazoles can be obtained via glycosylation of d-xylose with propargyl alcohol, followed by a click reaction and subsequent *N*-alkylation [43]. Another synthetic pathway involves selective bromination at the anomeric position with HBr, in which case the resulting halide can be further transformed into azide [24] or quaternized [23].

Billeci et al. [11,17] pioneered a third approach to IL synthesis using glucono-δ-lactone (Figure 3). A gluconamide derivative was formed through a reaction in methanol with either *N*,*N*-dimethylethylenediamine or *N*,*N*-dimethylpropylenediamine. The greatest advance related to this method is the elaboration of the purification procedure which does not involve column chromatography but rather a simple washing with an ethanol/ethyl acetate mixture, resulting in a 93% yield. The final ILs were obtained after quaternization of the corresponding amines with various alkyl halides such as 1-bromo/1-iodobutane, 1-bromooctane, 1-bromododecane, or 1-bromo-2-ethylhexane, where reported yields ranged from 83% to 97%. If applicable, this was followed by anion metathesis. The synthetic protocols used for these carbohydrate-based ILs adhere to the Principles of Green Chemistry [44], with atom economies equal to 100% in most cases and mass efficiencies/optimum efficiencies in the range of 69–96%.

Synthesis of ILs bearing sugar-derived anions is much more promising than converting sugars into cations, in terms of the required number of synthetic steps (Figure 4). However, the simple synthetic procedure is limited to acidic sugars which have a narrow degree of tunability of their properties, unlike ILs with sugar-derived cations. The synthetic procedure requires formation of the hydroxide of the desired cation using an ion exchange resin. The hydroxides utilized in carbohydrate-derived IL syntheses so far were prepared from *N,N,N’,N’*-tetramethylguanidine, tetraalkylammonium, tetraalkylphosphonium, or alkylimidazolium halides [12,18,22,37], which were further neutralized with commercially available acidic sugars (e.g., gluconic, glucuronic, or galacturonic acid). Synthesis of ILs with carbohydrate anions has similar complexity to the preparation of extensively applied cholinium-based ILs (i.e., 2 steps: ion exchange, followed by neutralization), yet it has been explored to a much lesser extent [45,46,47,48].

Another possibility to convert sugars into anions was presented by Gatard et al. (Figure 5) [49]. The synthetic procedure involved peracetylation of d-xylose, followed by reaction with corresponding ester (ethyl glycolate, methyl 6-hydroxyhexanoate or methyl 4-(hyroxymethyl)benzoate in the presence of BF_3_·Et_2_O. As formed compounds were deacetylated and neutralized with either tetrabutylammonium, tetrahexylammonium or tetrabutylphosphonium hydroxide, yielding corresponding ILs. This procedure offers more flexibility in physiochemical properties tuning by possible modifications of the carbohydrate unit, likewise in procedures that convert sugars into cations. 

## 3. Properties of Carbohydrate-Based ILs

**Figure 6:**
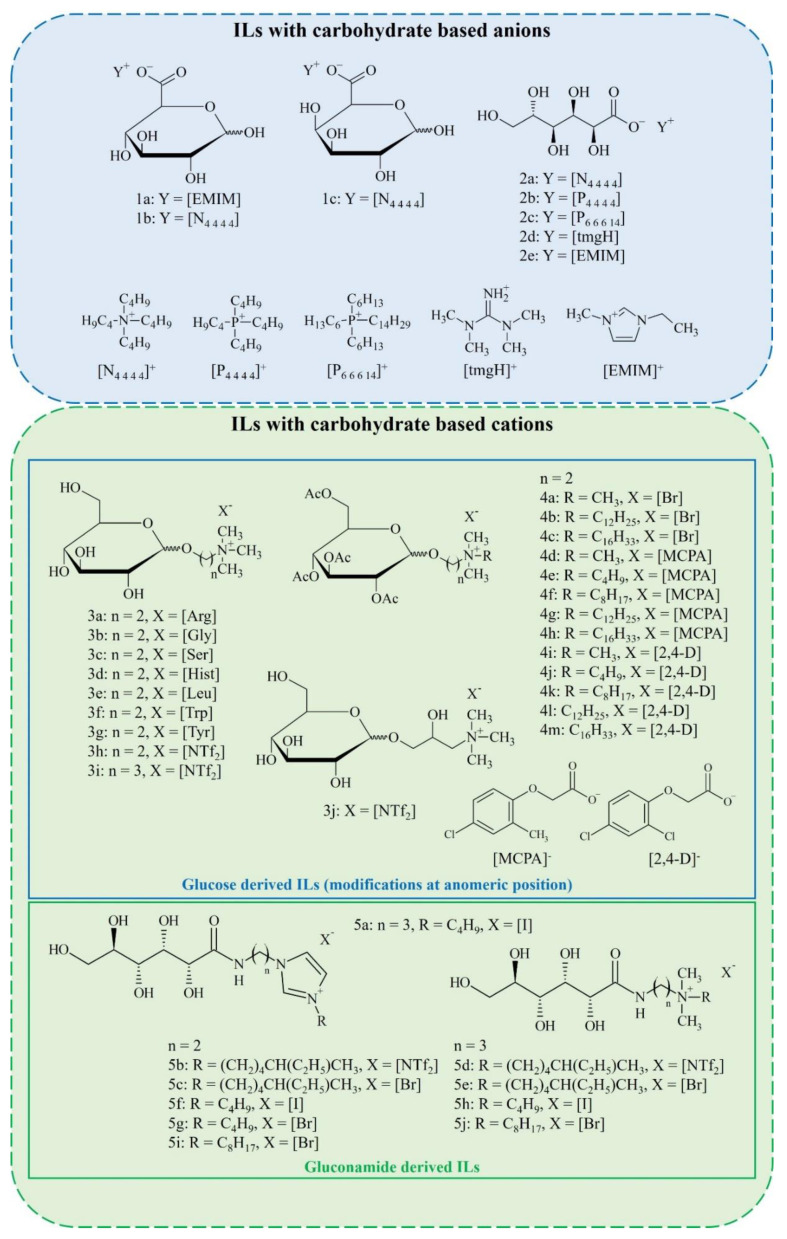

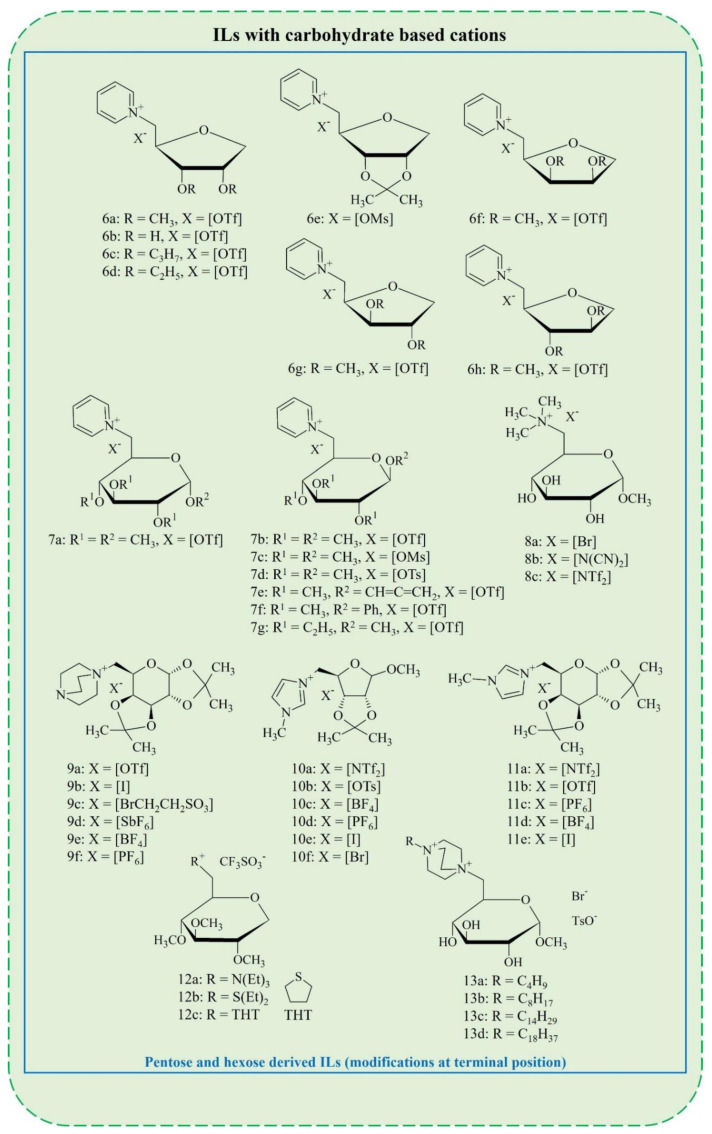

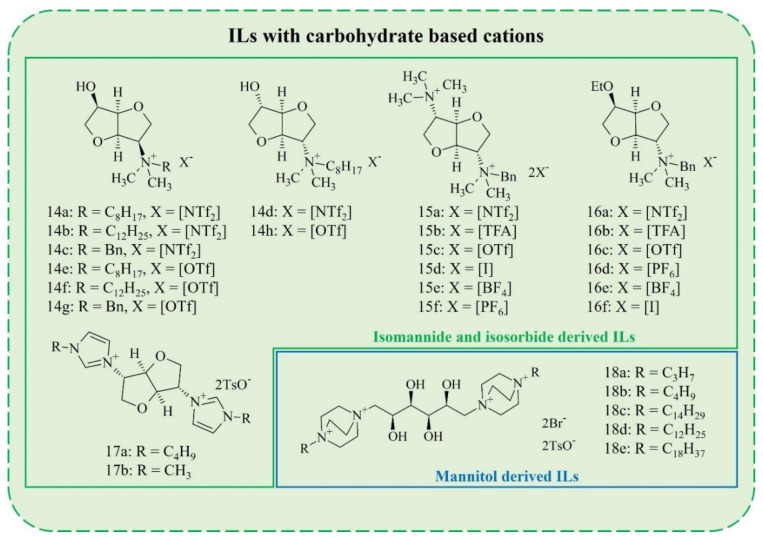
Structures of carbohydrate-derived ILs.

The structures of carbohydrate-derived ILs and salts significantly influence their potential environmental impacts as well as their physiochemical characteristics. These features are a result of (i) possessing carbohydrate units in either cationic or anionic forms and/or (ii) their functionalization which may include incorporation of alkyl chains of different lengths, alkyl spacers between carbohydrate moieties and quaternary ammonium groups, protection of hydroxyl groups, or quaternization with different amines. Herein, we compare over 100 carbohydrate-derived ILs and salts and discuss how their structures influence their biodegradability, toxicity, thermal properties, conductivity and viscosity in order to provide a comprehensive overview of the structure–properties relationships. Importantly, such comparisons can guide future design of sugar-rich systems for targeted applications. The structures of the ILs discussed in this review are presented in Figure 6, and their properties are collected in Table S1.

### 3.1. Biodegradation/Toxicity

Two of the key challenges currently faced in IL syntheses are related to using “green” synthetic techniques and minimizing the products’ ecological impacts. Many ILs are soluble in water, so their potential influence on the environment must be considered in case of any leakage or wastewater discharge leading to water or soil pollution [50,51]. According to Earle et al. [52], some ILs can be distilled at low pressure; therefore, atmospheric contamination cannot be completely neglected either, especially when ILs are used at elevated temperatures. Using natural and bio-renewable building blocks (i.e., organic acids, amino acids, or choline) frequently leads to biodegradable ILs [4,5]; however, in some cases, the resulting compounds still do not undergo complete biodegradation [53]. Additionally, as reviewed by Jordan and Gathergood [5], the biodegradability of the ILs is notably influenced by their chemical structures, where unbranched alkyl chains containing ester, formyl, carboxylic, or hydroxyl groups support biodegradation processes because they are readily hydrolyzed or oxidized. 

Ionic liquids with sugar moieties as their natural building blocks are rich in hydroxyl groups, which have been shown to enhance IL biodegradability, despite limited research in this area. Ferlin et al. [37], investigated a series of tetrabutylammonium ILs containing anions based on natural organic acids, and they reported that ILs derived from d-glucuronic (**1b**) and d-galacturonic (**1c**) acids demonstrate higher biodegradability than analogous ILs derived from l-lactic, l-tartaric, malonic, succinic, l-malonic, and pyruvic acids. Similarly, all of the bio-derived ILs investigated exhibited higher biodegradability in the Closed Bottle test (OECD 301D) than common tetrabutylammonium bromide and tetrabutylammonium hydroxide salts. However, the biodegradability of carbohydrate ILs is dependent on the length of the alkyl chain in the cation. In a series of quaternary ammonium salts based on d-glucose cation, where alkyl chains of various length were introduced (–CH_3_, –C_12_H_25_, –C_16_H_33_, **4a**, **4b**, **4c**, respectively), a derivative with the shortest alkyl chain (**4a**) was found to be readily biodegradable (74–83%), while **4b** with C12 was close to fulfill the ready biodegradability criterion (60%), reaching 57% [54]. In contrast, ILs with –C_16_H_33_ substituent (**4c**) showed a longer lag phase of 10 days, after which it started to degrade, not reaching a plateau during the experiment time though.

Combining carbohydrates with other bio-derived molecules (i.e., amino acids) has also been shown to yield readily biodegradable ILs (**3a**–**g**), which can be decomposed in 5–6 days in activated sludge [42]. This result highlights how carbohydrate-derived ILs can be used as alternatives to the cholinium family of ILs ([Ch]ILs), which have emerged as the most readily biodegradable ILs to date [5]. Besides cholinium amino acid-based ILs (AAILs) exhibit lower viscosity and more facile synthesis, although a green synthetic pathway for obtaining sugar-derived ILs has been reported recently [11], carbohydrates contain rich hydrogen bond structures which are favorable for certain applications. 

The toxic effects of the ILs and/or their metabolites on living organisms must be considered when evaluating their environmental impacts. In this context, ILs derived from naturally occurring compounds are also expected to be less toxic, with [Ch]ILs again being considered some of the most promising [55,56,57].

In addition to [Ch]ILs, carbohydrates have also gained attention as starting materials for synthesizing non-toxic and biocompatible ILs. Carbohydrate-derived ILs generally exhibit very low (eco)toxicity towards bacteria [37], fungi [37], human cancer cells [17], mouse cells [13,39], rat cells [54], and zebrafish eggs [17], indicating a promising biocompatible character.

Among the aforementioned tetrabutylammonium ILs combined with natural organic acids (i.e., d-glucuronic (**1b**), d-galacturonic (**1c**), l-lactic, l-tartaric, malonic, succinic, l-malic, and pyruvic acids), carbohydrate-derived ILs were determined to be the least toxic [37]. Interestingly, functionalization of conventional imidazolium ILs with sugar moieties decreased their toxicity or even removed it completely [13,17]. Hong et al. [13] further confirmed the positive effect of sugar moieties on toxicity by incorporating them into PILs. Incorporation of a quaternary ammonium salt into the polymer backbone functionalized with pendent sugar units revealed significantly decreased cytotoxic activity against mouse fibroblast cells (L929). 

However, Reiß et al. [39] reported that the toxicity of the ILs can depend on their concentration, and they demonstrated this relationship with a series of pentose- (**6a**–**h**) and d-glucopyranose-based ILs (**7a**–**g**). At a concentration of 0.1 M, almost all of the investigated ILs showed no cell viability; however, at 10 mM concentration, some differences were observed. Specifically, among pentoses-derived ILs, those based on ribose were the least toxic. Moreover, differences in toxicity between α- and β-anomers of glucose-derived ILs (**7a** and **7b**, respectively) were observed, with the IL based on the α-anomer demonstrating higher toxicity against mouse cells than the IL based on the β-anomer. 

The length of the carbon chain is another factor that can influence the cytotoxicity of ILs. This effect was discussed in aforementioned work by Erfurt et al. [54], where glucose-based ILs modified at anomeric position with various alkyl substituents (**4a**–**c**) were examined. Derivatives with the shortest alkyl chain (**4a**) showed the lowest cytotoxicity against rat leukemia cells, in fact it revealed no effect on the viability of cells up to 0.584 mM concentration. For derivatives **4b** and **4c,** the EC_50_ values were sufficiently lower: 0.085 for **4b** and 0.010 mM for **4c**, indicating an increase in cytotoxicity in the following order, **4a** < **4b** < **4c**. These results confirm that higher cytotoxicity corresponds to the higher hydrophobicity of investigated ILs. Surprisingly replacing [Br]^−^ anion with [NTf_2_]^−^ anion in **4c**, showed very little influence on the toxicity. The authors compared also investigated ILs with analogue dimethyl-phenyl-ammonium chloride compounds. The latter showed similar relation between cytotoxicity and hydrophobicity; however, replacing phenyl ring with hydrophilic d-glucose, significantly decreased their cytotoxicity [58].

Considering the cation/anion influence on the toxicity, the toxicity of ILs with cationic sugar moieties mostly depends on the anion. As reported for d-glucopyranose-derived ILs (**7b**–**d**), replacing [OTf]^−^ (**7b**) or [OTs]^−^ (**7d**) anions with [OMs]^−^ (**7c**) anions decreased the toxicity significantly. In contrast, for ILs where a carbohydrate moiety was incorporated as the anion, the toxicity of the systems was influenced primarily by the nature of the cation. For example, in gluconate ILs, those combined with phosphonium cations (**2b**,**2c**) tended to be more toxic than their ammonium (**2a**) or guanidinium (**2d**) analogues. Moreover, in agreement with previous reports, the increasing length of alkyl chains in a tetraalkylphosphonium cation can also negatively influence their toxicity [59,60].

The results presented in this section demonstrate that modification of conventional ILs with carbohydrate motifs may lead to decreased toxicity, thus further highlighting the significant potential of using carbohydrate units to fabricate biodegradable, non-toxic ILs. Building on these promising perspectives, further studies into the biological activity of carbohydrate-based ILs could indicate highly desirable applications in areas of chemistry as well as in biology and ecology [61].

### 3.2. Thermal Stability and Melting Point

The thermal properties of various ILs and salts determine their possible applications. ILs are frequently considered to be thermally stable because of their high decomposition temperatures (*T*_d_). However, data related to these properties are usually collected using ramped-temperature thermogravimetric analysis (TGA), and Del Sesto et al. [62] have reported that results obtained with this method can often be overestimated, even by hundreds of degrees, due to the high rates of temperature increase. The long-term stability of ILs can be evaluated using isothermal TGA (i.e., static TGA) measurements taken over a few hours. The *T*_d_ values obtained by such techniques are significantly lower than those measured with the ramped-temperature TGA method [63]. To the best of our knowledge, such experiments have not yet been conducted for carbohydrate-derived ILs, but they would be very useful to investigate caramelization of saccharides [64] which may also occur in saccharide-derived ILs.

Various structural features of the carbohydrate ILs may affect their thermal properties, including *T*_d_, melting point (*T*_m_), and glass transition temperature (*T*_g_). Here, we consider the influence of various cation modifications, including length of the alkyl chain on the quaternary ammonium group or alkyl spacer between carbohydrate moiety and quaternary ammonium group, additional functional/protecting groups, and the nature of both the cation and the anion.

#### 3.2.1. Anion

The type of anion in carbohydrate-derived ILs and salts as well as in their common imidazolium analogues corresponds to their nucleophilicity and basicity and significantly affect their thermal stability [65]. More nucleophilic and basic anions lead to less stable ILs and salts. This trend is clear for glucose-derived salts investigated by our group, with [Br]^−^, [NCN]^−^, and [NTf_2_]^−^ anions (**8a**, **8b**, and **8c**, respectively), where their thermal stabilities increased in the order, **8c** > **8b** > **8a**, corresponding to decreasing anion nucleophilicity [12,41]. 

This trend is also evidenced in the open chain gluconamide derivatives (**5b**, **5c**) investigated by Billeci et al., [17] where derivative **5b** with [NTf_2_]^−^ anions demonstrated higher thermal stability than **5c**, with [Br]^−^ anions. Interestingly, derivatives with longer alkyl spacers in their cations (i.e., **5d**, **5e**) followed the opposite tendency. The typical trend was maintained for isosorbide (generated via hydrogenation of glucose to sorbitol, followed by dehydration) derivatives, **14a**–**d** and **14e**–**h**, with [NTf_2_]^−^ and [OTf]^−^, respectively, where the latter exhibited higher thermal stability [34]. In halide-based gluconamide derivatives (**5f** and **5g**, with [I]^−^ and [Br]^−^, respectively), higher thermal stability was revealed for the derivative with the [I]^−^ anion, according to the decreasing basicity of halides ([F]^-^ > [Cl]^−^ > [Br]^−^ > [I]^−^). However, some deviations from this trend may occur. For example, Kaur et al. [40] reported a series of chiral salts derived from d-galactopyranose and DABCO (**9a**–**f**), with *T*_d_ values between 180 and 250 °C, which rise in the order, [OTf]^−^ < [I]^−^ < [BrCH_2_CH_2_SO_3_]^−^ < [SbF_6_]^−^ < [BF_4_]^−^ < [PF_6_]^−^.

Interestingly, a series of amino acid/carbohydrate-based ILs (**3a**–**g**) studied in our group revealed the profound influence of the amino acid structure on *T*_d_ [42]. Among the studied anions ([Arg]^−^, [Gly]^−^, [Ser]^−^, [His]^−^, [Leu]^−^, [Trp]^−^, and [Tyr]^−^), the thermal stability of the corresponding ILs increased with elongation of the amino acid side chain. The only exception was [Arg]^−^, which caused reduced stability of **3a**. 

Comparing the *T*_d_ values for derivatives where the carbohydrate moiety was transformed into an anion (e.g., cyclic glucuronate (**1b**) and open-chain gluconate (**2a**)), combined with a tetrabutylammonium cation, the latter exhibited higher thermal stability (*T*_d_ = 136 and 161.8 °C, respectively). Derivative **2a** also had a higher melting point and glass transition temperature than **1b** [17,37]. The same trend was observed for ILs with glucuronate and gluconate anions combined with [EMIM]^+^ cations. Specifically, *T*_d_ for the derivative with the glucuronate anion (**1a**) < 200 °C, whereas *T*_d_ for the gluconate anion derivative (**2e**) > 250 °C [12,22]. This is most likely due to the higher number of hydroxyl groups present in the gluconate anion (five, versus four in glucuronate) and the stronger H-bonding interactions. 

The type of anion also influences the *T*_m_ of carbohydrate-derived ILs and salts. Kumar et al. [30] prepared a series of dicationic salts derived from isomannide (**15a**–**f**), where the *T*_m_ values ranged from 60 to 251 °C, increasing in the following anion order: [NTf_2_]^−^ < [TFA]^−^ < [OTf]^−^ < [I]^−^ < [BF_4_]^−^ < [PF_6_]^−^. 

Certain carbohydrate salts with halide anions have exceptionally high *T*_m_ values, even higher than their analogues bearing [BF_4_]^−^ or [PF_6_]^−^ anions. In a series of chiral ammonium ILs and salts derived from isomannide (**16a**–**f**), the highest *T*_m_ (170 °C) was measured for the derivative bearing the [I]^−^ anion (**16f**) [31]. Derivatives with [NTf_2_]^−^ (**16a**) and [TFA]^−^ (**16b**) anions were liquids at room temperature; however, changing the anion to [OTf]^−^, [PF_6_]^−^, or [BF_4_]^−^ led to increases in their *T*_m_ values, to 80, 95, and 150 °C, respectively. This was also the case for d-ribose- (**10a**–**f**) and d-galactose- (**11a**–**e**) derived ILs and salts [26,66]. Specifically, d-ribose-based salts containing [NTf_2_]^−^, [OTs]^−^, or [BF_4_]^−^ anions were liquids at room temperature, while for the derivatives with [PF_6_]^−^, [I]^−^, or [Br]^−^ anions, melting points were observed, rising in the following order: [PF_6_]^−^ < [I]^−^ < [Br]^−^. The corresponding d-galactose derivatives were all solids with significantly higher *T*_m_ values.

#### 3.2.2. Cation

Both the origin and functionalization of an IL’s cation may affect the thermal properties of these sugar-based ILs. Poletti et al. [21] studied the influence of modifications at the C6 position of methyl-α-d-glucopyranoside using trimethylamine (**12a**), diethyl sulfide (**12b**), or tetrahydrothiophene (**12c**). Both **12a** and **12c** are solids at room temperature, with *T*_m_ = 137.5 and 110 °C, respectively. Additionally, **12c** showed an exothermic crystallization peak at 52.6 °C, in contrast to **12b**, which showed neither crystallization nor melting but formed a glass upon cooling to −53 °C. The thermal stability of these ILs increases in the order **12b** < **12c** < **12a**.

Reiß et al. [39] developed a series of pentose- (**6a**–**h**) and d-glucopyranose-based ILs (**7a**–**g**) which showed significant differences in thermal properties. Although direct comparisons are difficult to make because they have different functionalization at the anomeric centers, comparing pentose-derived ILs with glucoside-derived ILs possessing the same anions and protection of hydroxyl groups reveals that the pentose-derived ILs were mostly liquids at room temperature, while glucoside-derived ILs were mostly solids under the same conditions. Additionally, pentose-derived ILs have higher *T*_d_ values (297–345 °C), relative to glucoside-derived ILs (*T*_d_ = 205 to 250 °C). Among the pentose-derived ILs, thermal stability increases in the following order: d-lyxose < l-arabinose < d-xylose ≈ d-ribose. More exact comparisons can be made among glucoside-based products where differences in thermal properties were reported for α- and β-anomers. For example, the methyl-α-d-glucopyranoside-derived IL with [OTf]^−^ anions (**7a**) had a T_m_ of 95–100 °C, while the β-anomer (**7b**) was a liquid at room temperature. 

ILs where carbohydrate moieties were transformed into anions and combined with common alkylimidazolium, tetraalkylammonium, tetraalkylphosphonium, or *N*,*N*,*N*’,*N*’-tetramethylguanidium cations, demonstrate thermal stabilities in good correlation with the stability of cations reported for traditional ILs with non-carbohydrate-derived anions [12,17,37,63]. In a series of gluconate-based ILs with phosphonium, ammonium, or guanidium cations (**2a**–**d**), thermal stability depends on the nature of the cation and increases in the order, [tmgH]^+^ < [P_4 4 4 4_]^+^ < [N_4 4 4 4_]^+^ [17]. Moreover, their *T*_d_ values can be enhanced by elongation of the alkyl chain. This is evidenced by the fact that the derivative **2c**, with [P_6 6 6 14_]^+^ cations, exhibits the highest thermal stability, with *T*_d_ even higher than that of **2a**, with [N_4 4 4 4_]^+^ cations. Furthermore, as demonstrated for glucuronate-based ILs (**1a**, **1b**), the imidazolium salt (**1a**) showed higher thermal stability than the corresponding ammonium salt (**1b**) (i.e., [N_4 4 4 4_]^+^ < [Emim]^+^) [12]. This trend was also verified when imidazole was used as the functionalization moiety in a salt with a gluconamide-based cation (**5a**), which demonstrated higher thermal stability than the corresponding ammonium-based cation salt (**5h**) [17].

#### 3.2.3. Length of the Carbon Chain

The length of the carbon chain is another factor that can influence the thermal properties of carbohydrate-derived ILs and salts, where the carbon chain on the quaternary ammonium group and the spacer between the carbohydrate moiety and the quaternary ammonium group can be distinguished.

According to a report from Arellano et al. [67] investigating traditional imidazolium ILs, increasing the alkyl chain length strengthens Van der Waals intermolecular interactions which impact on the organization of the molecules in the sample, leading to increased thermal stability [68]. In contrast, increased chain lengths can also decrease the electrostatic interactions, thus causing lower thermal stability [69]. Moreover, it was shown that longer alkyl chain lengths in piperidinium ILs may increase the stability of the corresponding carbocations and carbon radicals, making them better leaving groups and thereby promoting decomposition [70]. In line with these observations, the thermal stability of carbohydrate-based ILs and salts show little dependence on elongations of alkyl chain length on the ammonium head. 

In a group of herbicidal ILs (HILs) derived from d-glucose, containing 4-chloro-2-methylphenoxyacetate anions and alkyl substituents at the ammonium head ranging from –CH_3_ to –C_16_H_33_ (**4d**–**h**), no obvious trend in thermal stability was observed [15]. However, the increase in thermal stability (i.e., C_1_ < C_8_ < C_4_ < C_16_ < C_12_) corresponded well with the analogous group of HILs with 2,4-dichlorophenoxyacetate anions (**4i**–**m**). One exception was derivative **4j**, with a C_4_ substituent, which demonstrated the lowest stability.

Comparison of the *T*_d_ values of isosorbide-derived ILs with C_8_ and C_12_ alkyl chains (**14a**, **14e**, and **14b**, **14f**, respectively) suggests that the length of the alkyl chain imposes very little influence on the thermal stability of this type of IL [34]. 

A more distinct influence of the alkyl chain length on the ammonium head could be determined considering the melting point data for investigated salts. For example, in the dicationic mannitol salts (**18a**–**e**), a systematic increase of *T*_m_ was observed to correlate with elongation of the chain length from –C_3_H_7_ to –C_18_H_37_ [25]. In addition, the isomannide-based salt with a **–**C_4_H_9_ alkyl chain (**17a**) has a higher melting point than its corresponding derivative with a methyl substituent (**17b**), which is an oil [32]. There is an observable, yet less profound influence of the alkyl chain length, which was also demonstrated for α-d-glucopyranoside derivatives functionalized with DABCO amine and quaternized with C_4_ to C_18_ alkyl chains (**13a**–**d**) **[25]**. 

The protection of hydroxyl groups by etherification provides a similar effect, as shown by glucose derivatives **7b** and **7g**, where hydroxyl groups have been methylated or ethylated, respectively [39]. Specifically, **7b** is a liquid at room temperature, while **7g** has a *T*_m_ of 118–120 °C. 

In terms of the alkyl spacer between the carbohydrate moiety and the quaternary ammonium group, thermal stability can be influenced by its linear or branched nature. In a group of glucono-based derivatives, the branching of the alkyl chain caused a decrease in *T*_d_ values (**5c**, **5i,** and **5e**, **5j**) [17]. Moreover, considering the length of the alkyl spacer, the thermal stability was dependent on both the nature of the anion and length of the alkyl chain. For glucose derivatives combined with [NTf_2_]^−^ anions and modified at the C1 position with ethoxy or propoxy chains (**3h**, **3i**), a slight increase in thermal stability was reported with elongation of the chain (∆T = 2 °C) [41]. This trend was also observed for glucono-based derivatives **5c** and **5e,** with ethyl and propyl linkers, respectively, combined with [Br]^−^ anions [17]. In contrast, the opposite trend (i.e., decrease of thermal stability with lengthening of the alkyl spacer) was reported for corresponding derivatives combined with [I]^−^ and [NTf_2_]^−^ anions (**5f**, **5h** and **5b**, **5d**, respectively). It is worth noting that, despite the opposite trend reported for derivatives with [NTf_2_]^−^ anions, the effect of lengthening the alkyl chain is minor (∆T = 3 °C), as demonstrated by comparing glucose derivatives **3h** and **3i** [41]. For halide derivatives with [I]^−^ (**5f, 5h**) and [Br]^−^ (**5c, 5e**) anions, the trend is noticeably more pronounced (∆T = 12 °C and ∆T = 27 °C, respectively) [17]. 

Concluding, conventional ILs exhibit higher thermal stabilities than most of carbohydrate derived ILs. It is certainly due to the easier degradation of carbohydrate motifs at elevated temperatures. 

Majority of ILs with hexose in the cation reveal *T*_d_ between 150 and 250 °C; however, additional stability can be achieved when combining sugar cation with [NTf_2_]^−^ anion. On the other hand, some pentose-derived ILs reveal higher thermal stability of 296–345 °C [39]. To compare, the conventional ILs with 1-ethyl-3-methylimidazolium cation and anions such as: [Cl]^−^, [Br]^−^, [I]^−^, [OTf]^−^, [OMs]^−^, [N(CN)_2_]^−^, [C(CN)_3_]^−^, [BF_4_]^−^, [PF_6_]^−^, [AsF_6_]^−^, [NTf_2_]^−^, [Beti]^−^, [EtSO_4_]^−^ reveal *T*_d_ ranging from 282 to 462 °C, [65], while ILs containing 1-butylpyridinium cation combined with: [Br]^−^, [BF_4_]^−^, [PF_6_]^−^, [NO_3_]^−^, [OTf]^−^, [N(CN)_2_]^−^, [HSO_4_]^−^, [H_2_PO_4_]^−^ reveal *T*_d_ ranging from 239 to 392 °C [69]. Simultaneously, by changing the anion in the IL with 1-ethyl-3-methylimidazolium cation into either gluconate or glucuronate, considerably lower thermal stabilities up to 250 °C are achieved [12,22,65]. These examples of pentose-based ILs show that certain carbohydrate ILs can be utilized in similar temperature ranges as the vastness of the conventional ILs, moreover, they are much less toxic.

### 3.3. Viscosity and Glass Transition Temperature

Viscosity (η) is a quantity describing a fluid’s internal flow resistance, and it is one of the key variables that determines an IL’s potential applications. This property is directly affected by different molecular interactions like repulsions, hydrogen bonding, short-range van der Waals interactions, and long-range electrostatic forces [71]. Therefore, carbohydrate-derived salts with significant hydrogen bonding capabilities commonly also exhibit relatively high viscosities, even orders of magnitude higher than their corresponding imidazolium, ammonium, or cholinium analogues [72]. Nevertheless, as reported by Billeci et al., it is still possible to design low viscosity carbohydrate-based ILs (e.g., η for **5g** = 106.3 mPa·s), which are promising solvents with potential industrial applications. Moreover, the problem of exceptionally high viscosities can be overcame in some applications by using mixture of IL and molecular solvent [73,74]. It is also worth noting that carbohydrate-based ILs have recently been recognized as promising materials for applications where low viscosity is not a demand (i.e., as organocatalysts or PILs with pendent sugar moieties). 

Currently, despite limited available data related to the viscosities of carbohydrate-based ILs, some structure–properties relationships can be inferred based on a survey published recently by Billeci et al. [17]. In a series of glucono-based ILs, changing the anion from [Br]^−^ to [I]^−^ induced a drastic increase in viscosity (i.e., from 106.3 mPa·s for **5g**, to 41,700 mPa·s for **5f**). Surprisingly, for these halides, the viscosity also increased with shortening of the alkyl spacer (from propyl to ethyl) between the carbohydrate moiety and the quaternary ammonium group (**5h** < **5f** and **5e** < **5c**). The trend is usually the opposite in the case of imidazolium ILs (i.e., increasing the length of the alkyl chains typically increases the viscosity through stronger van der Waals interactions [75]), and this opposite trend is also clear for glucono-based ILs with ethyl (**5b**) and propyl (**5d**) spacers and [NTf_2_]^−^ anions. Elongation of the alkyl chain on the ammonium head (from butyl in **5g** to octyl in **5c**) also led to more viscous liquids, similar to the effect of a smaller degree of branching (**5e** and **5j**, respectively) [17]. 

When combining carbohydrates with amino acids in ILs, the anion size also influences the viscosity. This relationship is evidenced by evaluating the effect of elongating the amino acid side chain. In general, the stronger intermolecular interactions accompanying such elongations resulted in increased viscosities of the derivatives **3a**–**g** [42]. 

The viscosity values are frequently reflected in the *T*_g_, which is the temperature at which reversible transition in amorphous materials occurs, from a hard, brittle, glass-like state to a viscous state [71,76]. Therefore, for ILs bearing the same anion, an increase of the *T*_g_ can be achieved by increasing the cation size, due to the expected stronger van der Waals interactions. These considerations relate to both cations and anions. Smaller anions with shorter alkyl chains commonly lead to lower *T*_g_ values [77,78]. This behavior is evidenced in a series of d-glucose-based ILs, where elongation of the alkyl spacer from ethyl to propyl (**3h** and **3i**, respectively) causes an increase in *T*_g_. 

Further increase in *T*_g_ is observed following introduction of an additional hydroxyl group on the hydrocarbon chain of the alkyl spacer in **3j** [41]. As expected, the additional hydroxyl group increased the overall strength of hydrogen bonding, which was then reflected in a higher *T*_g_ value. In addition, a decrease in *T*_g_ value was observed with greater branching of the alkyl side chain on the ammonium head in gluconamide-derived ILs (**5c** > **5i** and **5e** > **5j**) [17]. 

Considering the protection of hydroxyl groups of the sugar moieties, the opposite tendency in terms of alkyl chain length has been reported, i.e., an increase of *T*_g_ is observed with shortening of the alkyl chain length [39]. Similarly, it was reported for ribose-based ILs, that their *T*_g_ increased when changing propyl (**6c**) and ethyl (**6d**) ethers into methyl ethers (**6a**) [39]. 

The *T*_g_ of gluconamide-derived ILs increases with increasing nucleophilicity of the anion. For example, **5g** with [Br]^−^ anions have a reported *T*_g_ value of −46.6 °C, while for **5f**, [I]^−^ anions have *T*_g_ = −7.6 °C. Comparing *T*_g_ values of isosorbide-based ILs that contain the same cation and either [OTf]^−^ or [NTf_2_]^−^ anions, revealed that those with [OTf]^−^ anions had higher T_g_ values (i.e., **14a** < **14e**, **14c** < **14g**, and **14d** < **14h**) [34].

### 3.4. Conductivity

The conductivity of IL is one of the key parameters determining its use in electrochemical applications. As long as conventional ILs have been extensively studied as electrolytes for lithium batteries, fuel cells [79] or capacitors [80], very little research has been done in this area among carbohydrate ILs.

Billeci et al. [17] investigated the conductivity of gluconamide ILs (**5a**–**f**, **5h**–**j**) which were measured using [OMIM][BF_4_] as a standard. The conductivity of carbohydrate ILs ranged from 0.002 to 0.162 S/m, while for [OMIM][BF_4_] it was equal to 0.059 S/m. The lengthening of the alkyl spacer as well as its branching were the major factors influencing the decrease in the conductivity. Moreover, the degree of decrease was related to the anion coordination ability. The authors also compared the conductivity of **5c** with analogous compound, where the gluconamide motif was replaced with a hexanamide one. The latter showed a significantly higher ability of charge transport due to the lack of –OH groups that reveal strong hydrogen bonding ability leading to increased viscosity and, hence, lower conductivity. 

In another study, Chen et al. [38] investigated the conductivities of [C_n_MIM][Gluconate] (n = 2, 4, 6, 8, 10, 12 or 14) dissolved in various molecular solvents (water, ethanol and propyl alcohol). Again, regardless the solvent used, a decrease in conductivity was observed with the increase of alkyl chain length due to the lower mobility of such cations.

## 4. Applications and Future Perspectives 

**Figure 7:**
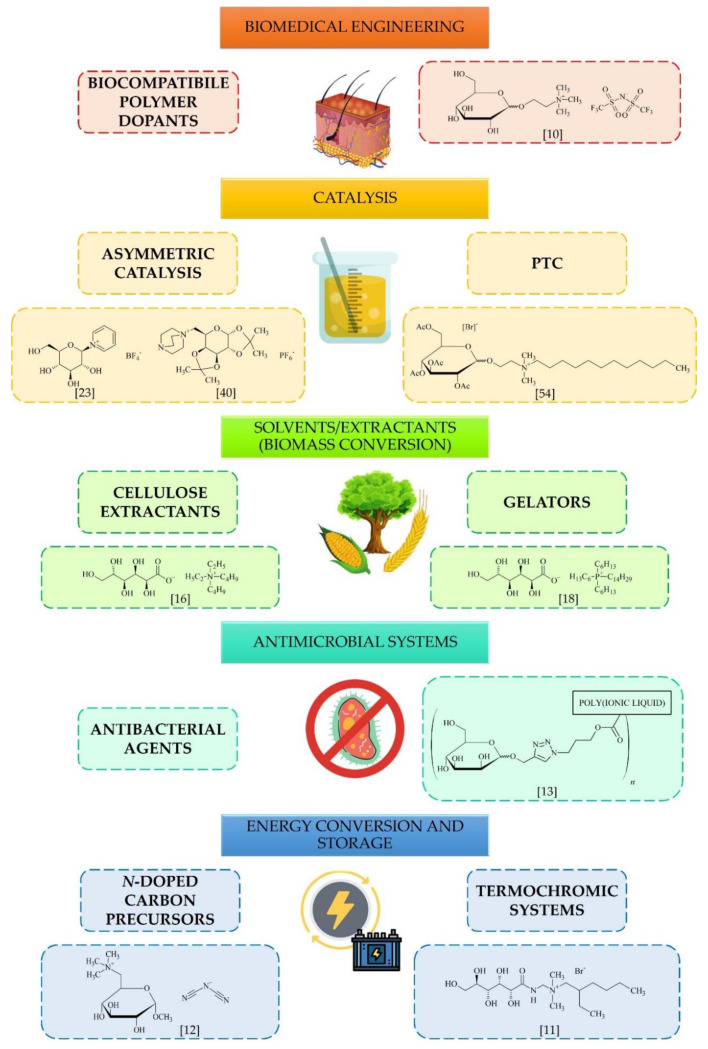
Applications and future perspectives for carbohydrate-derived ILs.

Due to versatile features, carbohydrate-derived ILs have been explored for a number of applications that span catalysis, biomedicine and ecology, energy conversion and storage or application as solvents (Figure 7). Here, we discuss some promising perspectives for their possible use.

### 4.1. Organocatalysts/Chiral Catalysts

Carbohydrates belong to the natural chiral pool, so their IL derivatives have primarily been studied as sustainable chiral solvents for applications in catalysis and materials synthesis. Since carbohydrate-based ILs were reviewed in the context of chiral building blocks for synthesis in 2017 [9], herein we focused on their potential as chiral catalysts based on examples from the most recently reported studies. 

Yuan et al. [23] used chiral glucose-containing pyridinium ionic liquids for a one-step asymmetric synthesis of Tröger’s base compounds which are used in supramolecular chemistry, DNA recognition, gas separation, medicine, or as promising catalysts in asymmetric reactions. The products were generated with high enantioselectivity (up to 84%) and efficiency (up to 83%) using a sugar-based IL as the catalyst and the solvent. 

Kaur and Chopra [40] investigated d-galactose- and DABCO-based ILs as chiral recognition agents for the enantiodifferentiation of sodium salts of Mosher’s acid. Moreover, chiral iodide salt was used as an organocatalyst in the asymmetric reduction of aromatic prochiral ketones. Secondary alcohols were obtained in moderate to high yields, although only low-to-moderate enantiomeric excess was achieved (5–17%). 

Sugar-based ILs have also been investigated as organocatalysts, making use of their task-specific properties originating from their rich hydrogen-bonding network structure. Specifically, Chrobok’s group [41] applied carbohydrate-based ILs to catalytically enhance Diels–Alder transformations, where catalytic amounts of ILs with respect to the dienophile were sufficient to obtain complete conversion (99% yield) and a high ratio of endo:exo isomers after several minutes. In fact, glucose-based ILs were as active as Yb(OTf)_3_, which is one of the most active metallic catalysts used for Diels–Alder reactions. The high number of hydroxyl groups in the cationic carbohydrate unit had a positive effect on both the kinetics and the selectivity of the reaction, as confirmed by comparative reactions conducted with ILs containing just one or two hydroxyl groups.

Very recently Chrobok’s group [54] presented also the application of glucose-derived ILs, modified with long alkyl chains on the quaternary ammonium group as PTC catalysts for the synthesis of 2-chloro-1,3-butadiene (chloroprene) in a two-phase system. The authors presented the influence of alkyl chain length of ammonium bromides on the conversion of 3,4-dichloro-1-butene, with the highest activity reported for IL bearing a C12 alkyl chain. The elaborated method, employing carbohydrate IL as PTC catalyst allowed for chloroprene formation with a high yield of >99% and 100% selectivity at room temperature in 1 h.

Carbohydrate-derived ILs have very good solubility in water, which can facilitate the separation of water-insoluble reaction products and regeneration of the IL for the next reaction cycle. ILs based on carbohydrates and amino acids were successfully utilized as catalysts for the Knoevenagel condensation reaction, where conversion values of 67–94% were achieved following reaction times as short as 15 min under exceptionally mild conditions using water as the solvent [42]. Moreover, AAILs have been intensively studied in the absorption of greenhouse gases due to the available amino group, suitable for efficient CO_2_, NO_x_, and SO_x_ capture. So far AAILs with common imidazolium [81,82], tetraalkylphosphonium [83], and tetraalkylammonium [84] cations as well as biocompatibile cholinium [85] have been shown as promising liquid absorbents. Nonetheless, this promising, sustainable approach still remains unexplored for carbohydrate based AAILs. 

### 4.2. Biomedicine and Ecology

The growing interest in the biological activity of ILs and their applications in the fields of biomedicine and ecology has initiated the development of bio-materials or drugs fabricated using ILs [61]. A considerable aspect of this newly emerging field is the establishment of a third generation of ILs, employing biodegradable and natural ions with known biological activities. Despite the fact that choline- and amino acid-based-ILs have been the primary types explored in biomedical areas to date, sugar-based ILs have the potential to become the next essential players. The antimicrobial activity and generally low toxicity of sugar-based ionic liquids have attracted the attention of researchers, leading to recent growth in the number of reports on their environmental impact, including toxicological aspects [13,17,37,39] and biodegradability [37,42].

Chen et al. [14] recently took advantage of the antibacterial properties of sugar-containing polymers, demonstrating that pyridinium-based PILs with pendent sugar units constitute a system with enhanced binding affinity and antibacterial activity against Gram-negative E. coli. The same group proposed fabrication of hybrid nanocomposites based on PILs containing quaternized amines and pendent sugar units with Fe_3_O_4_ nanoparticles as antibacterial agents for water treatment [13]. Incorporation of carbohydrate moieties into PILs has led to significant decrease in cytotoxicity towards mammalian cells and stronger antibacterial activity in comparison with unmodified PILs. In fact, the antibacterial and antifungal activities of polycationic carbohydrate-derived salts were already demonstrated a decade ago, and they also revealed promising activity as gels for topical disinfection [25]. 

Moreover, Billeci et al. [17] demonstrated that the glucono-based ILs could induce moderate or low antiproliferative effects and proved to be safe toward human erythrocytes in toxicity tests involving three different cancer cell lines and fish embryos. Toxic effects were detected only when unusually highly concentrated solutions were analyzed. Their research group also investigated functionalization of conventional imidazolium ILs with sugar moieties and found that these modifications decreased their toxicity. These very promising results may stimulate development of safer aromatic ILs and/or exclusively sugar-based IL systems for biomedical applications. 

Promising biocompatible character is also revealed when combining carbohydrates with other naturally occurring molecules in ILs. Recently, our group has investigated the combination of sugars with amino acids, which yielded readily biodegradable systems [42]. However, their biological activities have not yet been studied.

While investigating novel composites for biomedical engineering, Krukiewicz et al. [10] developed a polymer material, co-doped with a biodegradable, d-glucopyranoside-derived IL with conductive properties. Electrodeposition of poly(3,4-ethylenedioxythiophene) (PEDOT) in the presence of a carbohydrate IL (which also acts as the electrolyte) resulted in the formation of highly corrugated and structured polymer films with increased biocompatibility relative to undoped polymers. Thus, such composites represent promising candidates for applications as neural interfaces or tissue scaffolds.

Moreover, sugar-based ILs demonstrate herbicidal activity (as HILs) when combined with specific anions [15]. Pernak et al. in collaboration with Chrobok’s group, have reported that such ionic systems exhibit negligible vapor pressure, and their structures can be modified in order to control their water solubility, thus limiting their mobility in the environment.

### 4.3. Solvents (Biomass Conversion)

Ionic liquids have commonly been used as the reaction media for chemical transformations, taking advantage of their low volatility and high solubility of many organic precursors, but they can also be employed as extractants and dissolution media for processing biomass. 

In terms of reducing dependence on fossil fuel sources of hydrocarbons, the valorization of renewable biomass is highly desirable. In such processes, leftovers from food production or forestry activities can be utilized for manufacturing ILs, which can further be employed as regenerable solvents. Recently, the concept of a closed-loop biorefinery has been introduced, wherein biomass can be processed using ILs derived from biomass feedstock [7]. Considering this possibility, it would be beneficial to develop sugar-based ILs, suitable for breaking down hard-to-dissolve polysaccharides, because of their considerably lower toxicity relative to most other solvents used for biopolymer dissolution [7]. 

Javed et al. [16] used *N*,*N*-diethyl-*N*,*N*-dimethylammonium gluconate, easily prepared by neutralization of diethyl dimethyl ammonium hydroxide with gluconic acid, to extract cellulose from oil palm lignocellulosic biomass. By using this sugar-derived IL, 52% wt. of cellulose was extracted from the crude biomass, without any pre-treatment, within 30 min at 25 °C. Although conventional ILs have been intensely studied for potential polysaccharide dissolution purposes [86], sugar-based ILs have been investigated very little for this application. Additionally, carbohydrate-derived ILs commonly have high viscosities, but recently developed glucono-based derivatives with viscosities as low as 106.3 mPa·s make sugar-based ILs attractive solvents, with high hydrogen-bonding abilities and low environmental impacts.

Moreover, taking advantage of the strong coordinating abilities of hydroxyl groups, the potential use of this type of ILs as extractants should be explored. Recently, Billeci et al. [18] investigated gluconate-based ammonium and phosphonium salts, such as gelators to produce sugar-based gels with a thick network of hydrogen bonds, further used for desulfurization of fuels. Reported systems were able to remove approximately 70–80% of benzothiophene and dibenzothiophene, which are generally considered as refractory compounds in desulfurization.

### 4.4. Energy Conversion and Storage

Although conventional ILs have been widely studied in the context of their potential applications in electrochemistry, with a special focus on use as electrolytes [79], carbohydrate-based ILs have barely been investigated with regard to this approach. Nevertheless, researcher have begun exploring sugar-based ILs for applications related to energy conversion and storage.

Billeci et al. [11] exploited the coordinating properties of sugar-based ILs, reporting a thermochromic system that employed glucono-based ILs ligands to coordinate cobalt(II). The solution changed color from pink to blue upon heating/cooling in the range of 20–60 °C due to the change in the coordination geometry of the Co^2+^ ion with the hydroxyl groups present in the sugar moiety. This system was further incorporated into a polymer film and maintained its performance. Such stimuli-responsive “smart” systems have potential for energy storage applications like window glazing, because they use solar radiation energy with increased efficiency.

Ionic liquids have also been proposed as precursors for *N*-doped carbon materials, which have become desirable materials for energy conversion and storage, gas separation, or catalysis applications due to the fact of their excellent electric conductivity and chemical, mechanical, and thermal stability [87].

Recently our group has demonstrated that carbohydrate ILs can act as versatile *N*-doped carbon precursors that can simultaneously serve as the carbon source, *N*-dopant, and pore-generating agent [12]. Precise design of carbohydrate ILs at the molecular level allows scientists to finely tune the properties of the carbon materials in terms of yield, porosity, and nitrogen content. The resulting metal-free, nitrogen-doped carbon materials exhibited electrocatalytic activity toward oxygen reduction.

## 5. Summary and Outlook

Manufacturing ILs using abundant, inexpensive, and renewable carbohydrates represents a promising strategy for facing the challenge of sustainable chemical production with simultaneous depletion of fossil sources. As presented in this review, sugar-based ILs can be synthesized using various pathways, which have become more and more attractive from economic and sustainability perspectives. Although the purification of suitable intermediates is a bottleneck of carbohydrate IL synthesis, recently reported procedures that replace the column chromatography purification step with simple extraction using green organic solvents are examples of successful advancements toward development of green chemicals.

When considering applications that take advantage of the versatile features of carbohydrate-derived ILs, these derivatives can be used as alternatives to cholinium or amino acid derivatives, which are currently more commonly used in the synthesis of bio-ILs. The advantageous features of carbohydrate-derived ILs include their hydrogen bond-rich structure which endows these ionic liquids with high coordinating ability and solubility in polar solvents. Moreover, chirality, low toxicity, and high biodegradable potential of carbohydrate ILs make them attractive targets of research for catalytic, biomedical, and ecological applications. Overall, the spectrum of possible applications for sugar-based ILs has broadened considerably, to the point that they now demonstrate potential as solvents, extractants, or materials for energy conversion and storage.

One of the most attractive features of ILs is that their properties are amenable to tunability by selecting the specific cations, anions, side chains, or task-specific functional groups. The family of carbohydrate ILs and salts has only begun to be investigated, so there is a need to report more data describing the influence of structure on their (eco)toxicological properties. Overall, this will allow for more targeted design regarding this class of bio-ILs.

## Supplementary Materials

The following are available online at https://www.mdpi.com/1420-3049/25/14/3285/s1, Table S1: Thermal properties of carbohydrate ILs and salts.
