# Supplementary material for: Insights into the Properties and Potential Applications of Renewable Carbohydrate-Based Ionic Liquids: A Review
Source: Molecules. 2020 Jul 20;25(14):3285. doi: 10.3390/molecules25143285 (PMC7397332; doi:10.3390/molecules25143285)
Supplement: Supplementary file 1 [file molecules-25-03285-s001.pdf]

molecules

Supporting Information

Insights into the Properties and Potential Applications of Renewable Carbohydrate-Based Ionic Liquids—A Review

Bartłomiej Gaida [1] and Alina Brzęczek-Szafran [1,*]

[1] Department of Chemical Organic Technology and Petrochemistry, Silesian University of Technology, Krzywoustego 4, 44100 Gliwice, Poland
* Correspondence: alina.brzeczek-szafran@polsl.pl

Table S1. Thermal properties of carbohydrate ILs and salts. n.a. – not available, a – measured at 20 °C, b – measured in 70 °C, c – decomposition of 50% of the sample.

No.	Acronym	T_g [°C]	T_m [°C]	T_d [°C]	η [mPa s]	Ref.
1a	[EMIM][Glucuronate]	n.a.	Liquid	<200	n.a.	[1]
1b	[N$_{4444}$][Glucuronate]	-20.5	80-100	136	n.a.	[2]
1c	[N$_{4444}$][Galacturonate]	18.3	n.a.	n.a.	n.a.	[2]
2a	[N$_{4444}$][Gluconate]	0.4	120.0	161.8	n.a.	[3]
2b	[P$_{4444}$][Gluconate]	-21.8	80.2	155.0	n.a.	[3]
2c	[P$_{66614}$][Gluconate]	34.0	n.a.	161.8	n.a.	[3]
2d	[tmgH][Gluconate]	-24.1	n.a.	103.1	55870[a]	[3]
2e	[EMIM][Gluconate]	n.a.	Liquid	>250	n.a.	[4]
3a	[GlcO(CH$_2$)$_2$N$_{111}$][Arg]	-15	-	107	247626[b]	[5]
3b	[GlcO(CH$_2$)$_2$N$_{111}$][Gly]	-19	-	198	10196[b]	[5]
3c	[GlcO(CH$_2$)$_2$N$_{111}$][Ser]	-18	-	198	28645[b]	[5]
3d	[GlcO(CH$_2$)$_2$N$_{111}$][Hist]	-9	-	207	106930[b]	[5]
3e	[GlcO(CH$_2$)$_2$N$_{111}$][Leu]	4	28	208	359841[b]	[5]
3f	[GlcO(CH$_2$)$_2$N$_{111}$][Trp]	0	-	211	408284[b]	[5]
3g	[GlcO(CH$_2$)$_2$N$_{111}$][Tyr]	6	-	209	1476023[b]	[5]
3h	[GlcO(CH$_2$)$_2$N$_{111}$][NTf$_2$]	-30	n.a.	454.0[c]	n.a.	[6]
3i	[GlcO(CH$_2$)$_3$N$_{111}$][NTf$_2$]	-20	n.a.	456.0[c]	n.a.	[6]
3j	[GlcOCH$_2$CH(OH)CH$_2$N$_{111}$][NTf$_2$]	-14	n.a.	460.0[c]	n.a.	[6]
4a	[GlcO(CH$_2$)$_2$N$_{111}$][Br]	n.a.	n.a.	n.a.	n.a.	[7]
4b	[GlcO(CH$_2$)$_2$N$_{1112}$][Br]	n.a.	90-93	n.a.	n.a.	[8]
4c	[GlcO(CH$_2$)$_2$N$_{1116}$][Br]	n.a.	53-55	n.a.	n.a.	[8]
4d	[GlcO(CH$_2$)$_2$N$_{111}$][MCPA]	12	Wax	235	n.a.	[9]
4e	[GlcO(CH$_2$)$_2$N$_{114}$][MCPA]	20	Liquid	285	n.a.	[9]
4f	[GlcO(CH$_2$)$_2$N$_{118}$][MCPA]	10	Wax	259	n.a.	[9]
4g	[GlcO(CH$_2$)$_2$N$_{1112}$][MCPA]	13	Liquid	327	n.a.	[9]
4h	[GlcO(CH$_2$)$_2$N$_{1116}$][MCPA]	8	Wax	287	n.a.	[9]
4i	[GlcO(CH$_2$)$_2$N$_{111}$][2,4-D]	21	121	251	n.a.	[9]
4j	[GlcO(CH$_2$)$_2$N$_{114}$][2,4-D]	n.a.	134	247	n.a.	[9]
4k	[GlcO(CH$_2$)$_2$N$_{118}$][2,4-D]	22	Wax	279	n.a.	[9]
4l	[GlcO(CH$_2$)$_2$N$_{1112}$][2,4-D]	12	Wax	340	n.a.	[9]
4m	[GlcO(CH$_2$)$_2$N$_{1116}$][2,4-D]	15	Wax	296	n.a.	[9]
5a	[Im$_{3GA4}$][I]	-3.7	n.a.	197.3	5569[a]	[3]
5b	[N$_{112GA8}$][NTf$_2$]	-15.9	n.a.	191.1	17420[a]	[3]
5c	[N$_{112GA8}$][Br]	-16.6	n.a.	168.4	34880[a]	[3]
5d	[N$_{113GA8}$][NTf$_2$]	-16.2	n.a.	188.5	29030[a]	[3]
5e	[N$_{113GA8}$][Br]	-0.1	n.a.	195.1	18120[a]	[3]
5f	[N$_{112GA4}$][I]	-7.9	n.a.	210.3	41700[a]	[3]
5g	[N$_{112GA4}$][Br]	-46.6	n.a.	127.8	106.3[a]	[3]
5h	[N$_{113GA4}$][I]	-19.4	n.a.	197.7	31190[a]	[3]
5i	[N$_{112GA8L}$][Br]	-55.5	n.a.	200.6	21980[a]	[3]
5j	[N$_{113GA8L}$][Br]	-36.1	n.a.	200.6	n.a.	[3]
6a	[redRibMePyr][OTf]	-18	48-51	345	n.a.	[10]
6b	[redRibOHPyr][OTf]	-30	Liquid	297	n.a.	[10]
6c	[redRibPrPyr][OTf]	-27	Liquid	235	n.a.	[10]
6d	[redRibEtPyr][OTf]	-26	Liquid	316	n.a.	[10]
6e	[redRibIsoPyr][OMs]	n.a.	92-94	296	n.a.	[10]
6f	[redLyxMePyr][OTf]	-41	Liquid	325	n.a.	[10]
6g	[redXylMePyr][OTf]	-28	Liquid	345	n.a.	[10]

6h	[redAraMePyr][OTf]	-38	Liquid	340	n.a.	[10]
7a	[αMeGluMePyr][OTf]	n.a.	95-100	242	n.a.	[10]
7b	[βMeGluMePyr][OTf]	n.a.	Liquid	225	n.a.	[10]
7c	[βMeGluMePyr][OMs]	n.a.	60-63	250	n.a.	[10]
7d	[βMeGluMePyr][OTs]	n.a.	135-138	242	n.a.	[10]
7e	[βAllGluMePyr][OTf]	n.a.	66-70	205	n.a.	[10]
7f	[βPhGluMePyr][OTf]	n.a.	164-168	225	n.a.	[10]
7g	[βMeGluEtPyr][OTf]	n.a.	118-120	215	n.a.	[10]
8a	[N$_{1\,1\,1GLU}$][Br]	n.a.	n.a.	277[c]	n.a.	[1]
8b	[N$_{1\,1\,1GLU}$][N(CN)$_2$]	n.a.	124	281[c]	n.a.	[1]
8c	[N$_{1\,1\,1GLU}$][NTf$_2$]	n.a.	Liquid	429[c]	n.a.	[1]
9a	[GalDABCO][OTf]	n.a.	n.a.	178-182	n.a.	[11]
9b	[GalDABCO][I]	n.a.	n.a.	189-192	n.a.	[11]
9c	[GalDABCO][BrCH$_2$CH$_2$SO$_3$]	n.a.	n.a.	198-200	n.a.	[11]
9d	[GalDABCO][SbF$_6$]	n.a.	n.a.	202-206	n.a.	[11]
9e	[GalDABCO][BF$_4$]	n.a.	n.a.	222-224	n.a.	[11]
9f	[GalDABCO][PF$_6$]	n.a.	n.a.	249-252	n.a.	[11]
10a	[RibMIM][NTf$_2$]	n.a.	Liquid	n.a.	n.a.	[12]
10b	[RibMIM][OTs]	n.a.	Liquid	n.a.	n.a.	[12]
10c	[RibMIM][BF$_4$]	n.a.	Liquid	n.a.	n.a.	[12]
10d	[RibMIM][PF$_6$]	n.a.	96-98	n.a.	n.a.	[12]
10e	[RibMIM][I]	n.a.	124-126	n.a.	n.a.	[12]
10f	[RibMIM][Br]	n.a.	157-160	n.a.	n.a.	[12]
11a	[GalMIM][NTf$_2$]	n.a.	83-85	n.a.	n.a.	[12]
11b	[GalMIM][OTf]	n.a.	121-123	n.a.	n.a.	[12]
11c	[GalMIM]][PF$_6$]	n.a.	159-161	n.a.	n.a.	[12]
11d	[GalMIM]][BF$_4$]	n.a.	177-179	n.a.	n.a.	[12]
11e	[GalMIM][I]	n.a.	196-198	n.a.	n.a.	[13]
12a	[GluNEt$_3$][OTf]	n.a.	137.5	300.0	n.a.	[14]
12b	[GluSEt$_2$][OTf]	-53	n.a.	210.0	n.a.	[14]
12c	[GluTHT][OTf]	n.a.	110.0	250.0	n.a.	[14]
13a	[GluDABCOC$_4$][Br][OTs]	n.a.	102-107	n.a.	n.a.	[15]
13b	[GluDABCOC$_8$][Br][OTs]	n.a.	164-166	n.a.	n.a.	[15]
13c	[GluDABCOC$_{14}$][Br][OTs]	n.a.	129-130	n.a.	n.a.	[15]
13d	[GluDABCOC$_{18}$][Br][OTs]	n.a.	174-176	n.a.	n.a.	[15]
14a	[BnOIsoSN$_{1\,1\,8}$][NTf$_2$]	-49	n.a.	180	n.a.	[16]
14b	[BnOIsoSN$_{1\,1\,12}$][NTf$_2$]	-47	n.a.	180	n.a.	[16]
14c	[BnOIsoSN$_{1\,1\,Bn}$][NTf$_2$]	-29	n.a.	200	n.a.	[16]
14d	[HOIsoSN$_{1\,1\,8}$][NTf$_2$]	-35	n.a.	198	n.a.	[16]
14e	[BnOIsoSN$_{1\,1\,8}$][OTf]	-33	40	195	n.a.	[16]
14f	[BnOIsoSN$_{1\,1\,12}$][OTf]	-51	45	192	n.a.	[16]
14g	[BnOIsoSN$_{1\,1\,Bn}$][OTf]	-15	125	210	n.a.	[16]
14h	[HOIsoSN$_{1\,1\,8}$][OTf]	-15	42	204	n.a.	[16]
15a	[N$_{1\,1\,1}$IsoMN$_{1\,1\,Bn}$][NTf$_2$]$_2$	n.a.	60	n.a.	n.a.	[17]
15b	[N$_{1\,1\,1}$IsoMN$_{1\,1\,Bn}$][TFA]$_2$	n.a.	65	n.a.	n.a.	[17]
15c	[N$_{1\,1\,1}$IsoMN$_{1\,1\,Bn}$][OTf]$_2$	n.a.	75	n.a.	n.a.	[17]
15d	[N$_{1\,1\,1}$IsoMN$_{1\,1\,Bn}$][I]$_2$	n.a.	190	n.a.	n.a.	[17]
15e	[N$_{1\,1\,1}$IsoMN$_{1\,1\,Bn}$][BF$_4$]$_2$	n.a.	240	n.a.	n.a.	[17]
15f	[N$_{1\,1\,1}$IsoMN$_{1\,1\,Bn}$][PF$_6$]$_2$	n.a.	251	n.a.	n.a.	[17]
16a	[EtOIsoMN$_{1\,1\,Bn}$][NTf$_2$]	n.a.	Liquid	n.a.	n.a.	[18]
16b	[EtOIsoMN$_{1\,1\,Bn}$][TFA]	n.a.	Liquid	n.a.	n.a.	[18]

16c	[EtOIsoMN$_{1 1 Bn}$][OTf]	n.a.	80	n.a.	n.a.	[18]
16d	[EtOIsoMN$_{1 1 Bn}$][PF_6]	n.a.	95	n.a.	n.a.	[18]
16e	[EtOIsoMN$_{1 1 Bn}$][BF_4]	n.a.	150	n.a.	n.a.	[18]
16f	[EtOIsoMN$_{1 1 Bn}$][I]	n.a.	170	n.a.	n.a.	[18]
17a	[BimIsoMBim][OTs]$_2$	n.a.	150-152	n.a.	n.a.	[19]
17b	[MimIsoMMim][OTs]$_2$	n.a.	Liquid	n.a.	n.a.	[19]
18a	[DABCOC$_3$MannDABCOC$_3$][Br]$_2$[OTs]$_2$	n.a.	100-108	n.a.	n.a.	[15]
18b	[DABCOC$_4$MannDABCOC$_4$][Br]$_2$[OTs]$_2$	n.a.	109-112	n.a.	n.a.	[15]
18c	[DABCOC$_{14}$MannDABCOC$_{14}$][Br]$_2$[OTs]$_2$	n.a.	135-142	n.a.	n.a.	[15]
18d	[DABCOC$_{12}$MannDABCOC$_{12}$][Br]$_2$[OTs]$_2$	n.a.	158-168	n.a.	n.a.	[15]
18e	[DABCOC$_{18}$MannDABCOC$_{18}$][Br]$_2$[OTs]$_2$	n.a.	224-230	n.a.	n.a.	[15]

References

1. Brzęczek-Szafran, A.; Erfurt, K.; Blacha-Grzechnik, A.; Krzywiecki, M.; Boncel, S.; Chrobok, A. Carbohydrate Ionic Liquids and Salts as All-in-One Precursors for N-Doped Carbon. *ACS Sustain. Chem. Eng.* **2019**, *7*, 19880–19888.
2. Ferlin, N.; Courty, M.; Gatard, S.; Spulak, M.; Quilty, B.; Beadham, I.; Ghavre, M.; Haiß, A.; Kümmerer, K.; Gathergood, N.; et al. Biomass derived ionic liquids: Synthesis from natural organic acids, characterization, toxicity, biodegradation and use as solvents for catalytic hydrogenation processes. *Tetrahedron* **2013**, *69*, 6150–6161.
3. Billeci, F.; D'Anna, F.; Feroci, M.; Cancemi, P.; Feo, S.; Forlino, A.; Tonnelli, F.; Seddon, K.R.; Gunaratne, H.Q.N.; Plechkova, N.V. When Functionalization Becomes Useful: Ionic Liquids with a "sweet" Appended Moiety Demonstrate Drastically Reduced Toxicological Effects. *ACS Sustain. Chem. Eng.* **2020**, *8*, 926–938.
4. Costa, A.; Forte, A.; Zalewska, K.; Tiago, G.; Petrovski, Z.; Branco, L.C. Novel biocompatible ionic liquids based on gluconate anion. *Green Chem. Lett. Rev.* **2015**, *8*, 8–12.
5. Brzęczek-Szafran, A.; Więcek, P.; Guzik, M.; Chrobok, A. Combining amino acids and carbohydrates into readily biodegradable, task specific ionic liquids. *RSC Adv.* **2020**, *10*, 18355–18359.
6. Erfurt, K.; Wandzik, I.; Walczak, K.; Matuszek, K.; Chrobok, A. Hydrogen-bond-rich ionic liquids as effective organocatalysts for Diels-Alder reactions. *Green Chem.* **2014**, *16*, 3508–3514.
7. Erfurt, K.; Markiewicz, M.; Siewniak, A.; Zalewski, M.; Stolte, S.; Chrobok, A. Biodegradable surface active D-glucose based quaternary ammonium ionic liquids in the solventless synthesis of chloroprene. *ACS Sustain. Chem. Eng.* **2020**.
8. Quagliotto, P.; Viscardi, G.; Barolo, C.; Angelo, D.D.; Barni, E.; Compari, C.; Duce, E.; Fisicaro, E. Synthesis and Properties of New Glucocationic Surfactants : Model Structures for Marking Cationic Surfactants with Carbohydrates. **2005**, *70*, 9857–9866.
9. Pernak, J.; Czerniak, K.; Biedziak, A.; Marcinkowska, K.; Praczyk, T.; Erfurt, K.; Chrobok, A. Herbicidal ionic liquids derived from renewable sources. *RSC Adv.* **2016**, *6*, 52781–52789.
10. Reiß, M.; Brietzke, A.; Eickner, T.; Stein, F.; Villinger, A.; Vogel, C.; Kragl, U.; Jopp, S. Synthesis of novel carbohydrate based pyridinium ionic liquids and cytotoxicity of ionic liquids for mammalian cells. *RSC Adv.* **2020**, *10*, 14299–14304.
11. Kaur, N.; Chopra, H.K. Synthesis and applications of carbohydrate based chiral ionic liquids as chiral recognition agents and organocatalysts. *J. Mol. Liq.* **2020**, *298*, 111994.
12. Jayachandra, R.; Reddy, S.R. Synthesis of D-ribose and D-galactose Derived Chiral Ionic Liquids as Recyclable Chiral Solvent for Michael Addition Reaction. *Trends Carbohydr. Res.* **2015**, *7*, 60–67.
13. Jayachandra, R.; Lakshmipathy, R.; Reddy, S.R. Hydrophobic d-galactose based ionic liquid for the sequestration of Pb2 + ions from aqueous solution. *J. Mol. Liq.* **2016**, *219*, 1172–1178.
14. Poletti, L.; Chiappe, C.; Lay, L.; Pieraccini, D.; Polito, L.; Russo, G. Glucose-derived ionic liquids: Exploring low-cost sources for novel chiral solvents. *Green Chem.* **2007**, *9*, 337–341.
15. Thomas, M.; Montenegro, D.; Castaño, A.; Friedman, L.; Leb, J.; Huang, M.L.; Rothman, L.; Lee, H.; Capodiferro, C.; Ambinder, D.; et al. Polycations. 17. Synthesis and properties of polycationic derivatives of carbohydrates. *Carbohydr. Res.* **2009**, *344*, 1620–1627.

16. Van Buu, O.N.; Aupoix, A.; Hong, N.D.T.; Vo-Thanh, G. Chiral ionic liquids derived from isosorbide: Synthesis, properties and applications in asymmetric synthesis. *New J. Chem.* **2009**, *33*, 2060–2072.
17. Kumar, V.; Olsen, C.E.; Schäffer, S.J.C.; Parmar, V.S.; Malhotra, S.V. Synthesis and applications of novel bis(ammonium) chiral ionic liquids derived from isomannide. *Org. Lett.* **2007**, *9*, 3905–3908.
18. Kumar, V.; Pei, C.; Olsen, C.E.; Schäffer, S.J.C.; Parmar, V.S.; Malhotra, S.V. Novel carbohydrate-based chiral ammonium ionic liquids derived from isomannide. *Tetrahedron Asymmetry* **2008**, *19*, 664–671.
19. Gomes Da Silva, M.D.R.; Pereira, M.M.A. New chiral imidazolium ionic liquids from isomannide. *Carbohydr. Res.* **2011**, *346*, 197–202.

© 2020 by the authors. Licensee MDPI, Basel, Switzerland. This article is an open access article distributed under the terms and conditions of the Creative Commons Attribution (CC BY) license (http://creativecommons.org/licenses/by/4.0/).
